# Mainstreamed genetic testing of breast cancer patients in two hospitals in South Eastern Norway

**DOI:** 10.1007/s10689-020-00160-x

**Published:** 2020-01-30

**Authors:** Eli Marie Grindedal, Kjersti Jørgensen, Pernilla Olsson, Berit Gravdehaug, Hilde Lurås, Ellen Schlichting, Tone Vamre, Teresia Wangensteen, Cecilie Heramb, Lovise Mæhle

**Affiliations:** 1 Department of Medical Genetics, Oslo University Hospital, Oslo, Norway; 2 Department of Surgery, Section of Breast and Endocrine Surgery, Innlandet Hospital, Hamar, Norway; 3 Department of Breast and Endocrine Surgery, Akershus University Hospital, Lørenskog, Norway; 4 Health Services Research Unit, Akershus University Hospital, Lørenskog, Norway; 5 Institute of Clinical Medicine, University of Oslo, Oslo, Norway; 6 Department of Oncology, Section of Breast- and Endocrine Surgery, Oslo University Hospital, Oslo, Norway

**Keywords:** Genetic testing, BRCA, Breast cancer, Mainstreaming cancer genetics

## Abstract

Studies have shown that a significant number of eligible breast cancer patients are not offered genetic testing or referral to genetic counseling. To increase access to genetic testing in South Eastern Norway, testing has since 2014 been offered directly to breast cancer patients by surgeons and oncologists. This practice is termed “mainstreamed genetic testing”. The aim of this study was to investigate to what extent patients in South Eastern Norway are offered testing. Three hundred and sixty one patients diagnosed in 2016 and 2017 at one regional and one university hospital in South Eastern Norway were included. Data on whether the patients fulfilled the criteria, whether they had been offered testing and if they were tested were collected. In total, 26.6% (96/361) fulfilled the criteria for testing. Seventy five percent (69/92) of these were offered testing, and 71.7% (66/92) were tested. At the university hospital, 90.2% (37/41) of eligible patients were offered testing, and at the regional hospital 62.7% (32/51). Fifty two percent (12/23) of eligible patient not offered testing were younger than 50 years at time of diagnosis. As many as 95.4% (125/131) of all patients who were offered testing, wanted to be tested. The majority of patients who fulfilled the criteria were offered testing, supporting the practice of mainstreamed genetic testing. There were nevertheless differences in rates of testing between the hospitals that affected all groups of patients, indicating that genetic testing may not be equally accessible to all patients. We suggest that efforts should be made to increase awareness and improve routines for genetic testing of breast cancer patients in Norway.

## Background

Germline pathogenic variants in *BRCA1* and *BRCA2* are associated with a high lifetime risk of breast and ovarian cancer [1–3]. Identification of a pathogenic variant in one of these genes in a woman diagnosed with breast cancer (BC) provides critical information for treatment decisions for her current cancer [4–9]. In addition, future breast and ovarian cancer may be prevented through risk-reducing mastectomy and salpingo-oophorectomy in herself and her relatives who may also carry the variant [10–12]. Genetic testing of these two genes is therefore increasingly offered to BC patients at time of diagnosis or during treatment.

In most countries, genetic testing is only offered to BC patients with an a priori high risk of being a carrier of a pathogenic variant, either because they have BC at a young age (below 50 years), triple negative BC (TNBC), or because they have a family history of breast and/or ovarian cancer. The Norwegian Breast Cancer Group (NBCG) has developed criteria for *BRCA* testing of BC patients based on such risk factors (see Table 1) [13]. Similar guidelines have been developed in other countries [14, 15]. However, several studies have demonstrated that a significant number of BC patients who fulfill these criteria are neither offered genetic testing nor referred to genetic counseling [16–20].

**Table 1:** NBCG criteria for diagnostic genetic testing of breast cancer patients in 2016

Woman with breast cancer < 50 years^a^	
Two close relatives^a^ with breast cancer, mean age < 55 years	
Three close relatives^a^ with breast cancer at any age	
Male breast cancer	
Woman with bilateral breast cancer < 60 years	
Woman with breast cancer and a close relative with ovarian cancer^b^	
Woman with breast cancer and a close relative with prostate cancer < 55 years^b^	
Woman with ovarian cancer at any age	
Woman with triple negative breast cancer < 60 years (as recommended by the National Comprehensive Cancer Network, USA)^c^	

^a^In 2018, the age limit for testing was raised to 60 years

^b^Close relative is a first degree relative, or a second degree relative through a man

^c^Included in the criteria from 2017

We have recently estimated that about 39% of all BC patients in the South-Eastern Norway Regional Health Authority (hereafter called South Eastern Norway) were tested in 2014 and 2015 [21]. However, we do not know how many of the patients who fulfilled the criteria that were offered testing. Based on the previous studies on rates of genetic testing, we suspect that also in Norway there may be BC patients eligible for testing according to the NBCG criteria who are not offered testing. We also suspect that the rate of genetic testing of BC patients may be higher in South Eastern Norway than the previous studies have shown. One explanation might be that many of the previous studies report observations from before 2010, and the awareness and availability of genetic testing has increased significantly during the last 5 years. Another explanation may be that in South Eastern Norway, genetic testing is offered directly to BC patients by the treating surgeon or oncologist as part of regular surgical and/or oncological health care, a model called “mainstreamed genetic testing” [22]. The patient is only referred to genetic counseling if a pathogenic variant or a variant of unknown clinical significance (VUS) is detected. This is different from the traditional model where genetic tests are ordered by specialists in medical genetics or genetic counselors and only after genetic counseling. It has been argued that the traditional model contributes to keeping rates of genetic testing low [23].

The role of genetic testing in treatment of BC and other cancers will increase in the coming years. This is both due to the decreasing costs associated with such testing, the increasing knowledge of different genes associated with heritable cancer risk, and new opportunities for personalized treatment for hereditary tumors. Knowledge on how the health service of genetic testing is practiced is therefore needed to ensure that testing is equally available to all eligible patients across hospitals and health regions.

The aim of this study was to explore to what extent genetic testing of BC patients is provided at two hospitals in South Eastern Norway, one regional and one university hospital. In both hospitals, genetic testing is offered directly to the patient by the treating surgeon or oncologist: i.e. within a “mainstreaming genetic testing” model. More specifically, we investigated how many BC patients that were offered genetic testing, and how many of them that wanted to be tested. We also explored how many of the BC patients who fulfilled the NBCG criteria were offered testing, and the clinical characteristics such as age at BC diagnosis and family history of cancer of those who fulfilled the criteria that were not offered testing. Data were collected for patients diagnosed during the first half of 2016 and 2017.

## Methods

### Mainstreamed genetic testing in South Eastern Norway

All genetic analyses for hereditary cancer in South Eastern Norway are done at Department of Medical Genetics (DMG) at Oslo University Hospital (OUH). In 2014, surgical departments at all hospitals in South Eastern Norway and DMG agreed that the treating surgeon or oncologist could offer newly diagnosed BC patients who fulfill the criteria, diagnostic genetic testing of the *BRCA* genes without referring the patient to genetic counseling prior to ordering the test. The aim of this procedural change was both to increase access to genetic testing for BC patients and to obtain test results in a time that allowed the surgeon or oncologist to incorporate the results into treatment decisions. DMG developed written information and consent forms, and specialists in Medical Genetics, genetic counselors and molecular geneticists from DMG held informational meetings at all hospitals. Apart from these meetings, surgeons and oncologists did not receive any specific training in medical genetics. Patients who wanted to be referred to genetic counseling before testing could still be referred. Patients who tested positive for a pathogenic variant or a VUS would all be referred to genetic counseling. The patient’s family history of cancer should be recorded when she is admitted to the hospital for treatment. The treating physician only offers testing of *BRCA1* and *BRCA2.* Patients who had a normal *BRCA* test but had a family history of cancer that indicated either testing of other genes, and/or that she or her relatives should be recommended follow up for familial risk of BC, would also be referred to genetic counseling.

### Patients

The two hospitals involved in the study were Akershus University Hospital (Ahus) and Innlandet Hospital Trust (IH). Ahus serves a population of 500.000 and IH a population of 380.000.

All patients diagnosed with invasive BC between 1st of January and 30th of June in 2016 and 2017 were identified, 303 from Ahus and 256 from IH. These two time periods were chosen to uncover a potential increase in the use of genetic testing from 2016 to 2017, as there was a small change in the criteria in 2017 (see Table 1). All patients were sent an information letter and a consent form to give access to their hospital records. One hundred and ninety nine BC patients from Ahus and 162 from IH signed the consent form, giving a response rate of 65.7% and 63.3% for the two hospitals respectively. Mean age was similar for the two groups, 61.1 (range 33–92) for Ahus and 60.4 (range 28–86) for IH. The distribution of patients in different age groups was also similar for the two hospitals. See Table 2 for a description of the cohorts.

**Table 2:** Description of cohorts

	Innlandet hospital (*n* = 162)	Ahus (*n* = 199)	Combined (*n* = 361)
Mean age	60.4 (range 28–86)	61.1 (range 33–92)	60.8 (range 28–92)
Age cohorts			
20–29	1 (0.6%)	–	1 (0.3%)
30–39	5 (3.1%)	4 (2.0%)	9 (2.5%)
40–49	23 (14.2%)	27 (13.6%)	50 (13.9%)
50–59	48 (29.6%)	52 (26.1%)	100 (27.7%)
60–69	55 (34.0%)	77 (38.7%)	132 (36.6%)
70-	30 (18.5%)	39 (19.6%)	69 (19.1%)

### Methods

We collected data on age at diagnosis, whether the patient had bilateral BC and whether the tumor was triple negative (ER, PR and HER2 negative) from the Electronic Patient Record (EPR). In addition, information was collected on whether the patient had been asked about their family history of cancer, whether they had a family history of cancer and if yes, what type of cancers. The patients were then scored according to the NBCG criteria used at time of diagnosis (see Table 1). When information in the EPR was not sufficient to score the patient according to the criteria, we registered that it was uncertain whether the patient fulfilled them. Finally, data was collected on whether or not the patient had been offered genetic testing, if yes by whom, and whether the patient had been tested. We also registered whether it was the patient who had asked for the test.The data were registered in a web based form and stored at the Service for Sensitive Data (TSD, University of Oslo). No demographic data like education level, employment status, ethnic background, marital or familial status were collected.

In the consent form the patients could tick off that they wanted to be contacted if they were eligible for genetic testing according to the criteria. Patients that ticked off the box and had not been tested before, but fulfilled the criteria in use in 2018, were contacted and offered testing. Patients who could not be scored according to the criteria in use in 2018 were contacted for evaluation of family history and offered testing if they fulfilled the criteria.

### Non-responders

Ninety four BC patients at IH (36.7%) and 104 (34.3%) at Ahus did not sign the consent form. Their mean age was 64 and 60.6 years respectively. This is similar to the mean age of the patients included in the study. No other demographic information was collected on the patients that did not sign the consent form.

### Statistics

We report descriptive statistics of our findings for the two hospitals separately and combined, and present percentages of patients falling into the different categories investigated. Due to the limited size of the datasets, no statistical comparison of the two hospitals was made. The results for 2016 and 2017 were similar and were therefore combined in the analyses.

### Ethics

The research project was evaluated by the Regional Committees for Medical and Health Research Ethics. They defined it as a quality of care study, and therefore outside of their mandate. The study has been approved by the data protection officers at Oslo University Hospital (OUH), Ahus and IH. Informed consent was obtained from all individual participants included in the study.

## Results

Results regarding use of genetic testing were similar for the two time periods investigated (first half of 2016 and first half of 2017), and were therefore combined.

### Genetic testing of all patients

In one of the medical records from IH and four from Ahus it was noted that the patient had been tested prior to their BC diagnosis. These five patients were not offered a new test during diagnosis and treatment of their BC. Excluding them from the denominator, 131 of 356 patients (36.8%) had been tested, 48/161 (29.8%) at IH and 83/195 (42.6%) at Ahus. Of the 131 who were offered testing, 125 wanted to be tested (95.4%). The test had been requested by the surgeon prior to surgery in 71/125 (56.8%) of patients, and by the oncologist in 53/125 (42.4%). See Table 3.

**Table 3:** Genetic testing of all patients and evaluation of criteria

	Innlandet hospital (*n* = 162)	Ahus (*n* = 199)	Combined (*n* = 361)
Genetic testing			
Offered genetic testing	*n* = 161^a^ 48 (29.8%)	*n* = 195^a^ 83 (42.6%)	*n* = 356^a^ 131 (36.8%)
Tested	*n* = 161^a^ 45 (27.8%)	*n* = 195^a^ 80 (40.2%)	*n* = 356^a^ 125 (34.6%)
Uptake of genetic testing	*n* = 48 45 (93.8%)	*n* = 83 80 (96.4%)	*n* = 131 125 (95.4%)
Test ordered by	*n* = 45	*n* = 80	*n* = 125
Surgeon	23 (51.1%)	48 (60%)	71 (56.8%)
Oncologist	22 (48.9%)	31 (38.8%)	53 (42.4%)
Other		1 (1.3%)	1 (0.8%)
Evaluation of family history			
Asked about family history of cancer	*n* = 162 126 (77.8%)	*n* = 199 189 (95.0%)	*n* = 361 315 (87.3%)
Reported family history of breast and/or ovarian cancer	*n* = 126 58 (46.0%)	*n* = 189 66 (34.9%)	*n* = 315 124 (39.4%)
Criteria fulfilled			
BC < 50 years	*n* = 162 29 (17.9%)	*n* = 199 31 (15.6%)	*n* = 361 60 (16.6%)
Bilateral BC < 60 years	*n* = 162 2 (1.2%)	*n* = 199 2 (1%)	*n* = 361 4 (1.1%)
TNBC < 60 years	*n* = 162 3 (1.9%)	*n* = 199 3 (1.5%)	*n* = 361 6 (1.7%)
Male breast cancer	–	*n* = 199 2 (1.0%)	*n* = 361 2 (0.5%)
Family history of BC and/or OC^b^	*n* = 126 18 (14.3%)	*n* = 189 6 (3.2%)	*n* = 315 24 (7.6%)

^a^Excluded patients who had been tested prior to their breast cancer diagnosis

^**b**^These patients were 50 years or older at time of diagnosis, and did not fulfill any of the other criteria (TNBC < 60 years, bilateral B < 60 years or male BC)

### Fulfillment of criteria

Most of the patients who fulfilled the criteria for testing, did so due to young age at diagnosis (below 50 years): Twenty nine out of 162 (17.9%) patients at IH and 31/199 (15.6%) at Ahus. It was noted in the medical records of 126/162 (77.8%) patients at IH and 189/199 (95.0%) at Ahus that they had been asked about their family history. Of these, 18/126 (14.3%) patients at IH and 6/189 (3.2%) at Ahus fulfilled the criteria due to family history of cancer only (i.e. they did not have BC < 50 years/TNBC < 60 years/bilateral BC < 60 years/male BC). See Table 3.

### Genetic testing according to the NBCG criteria

**Fig. 1:**
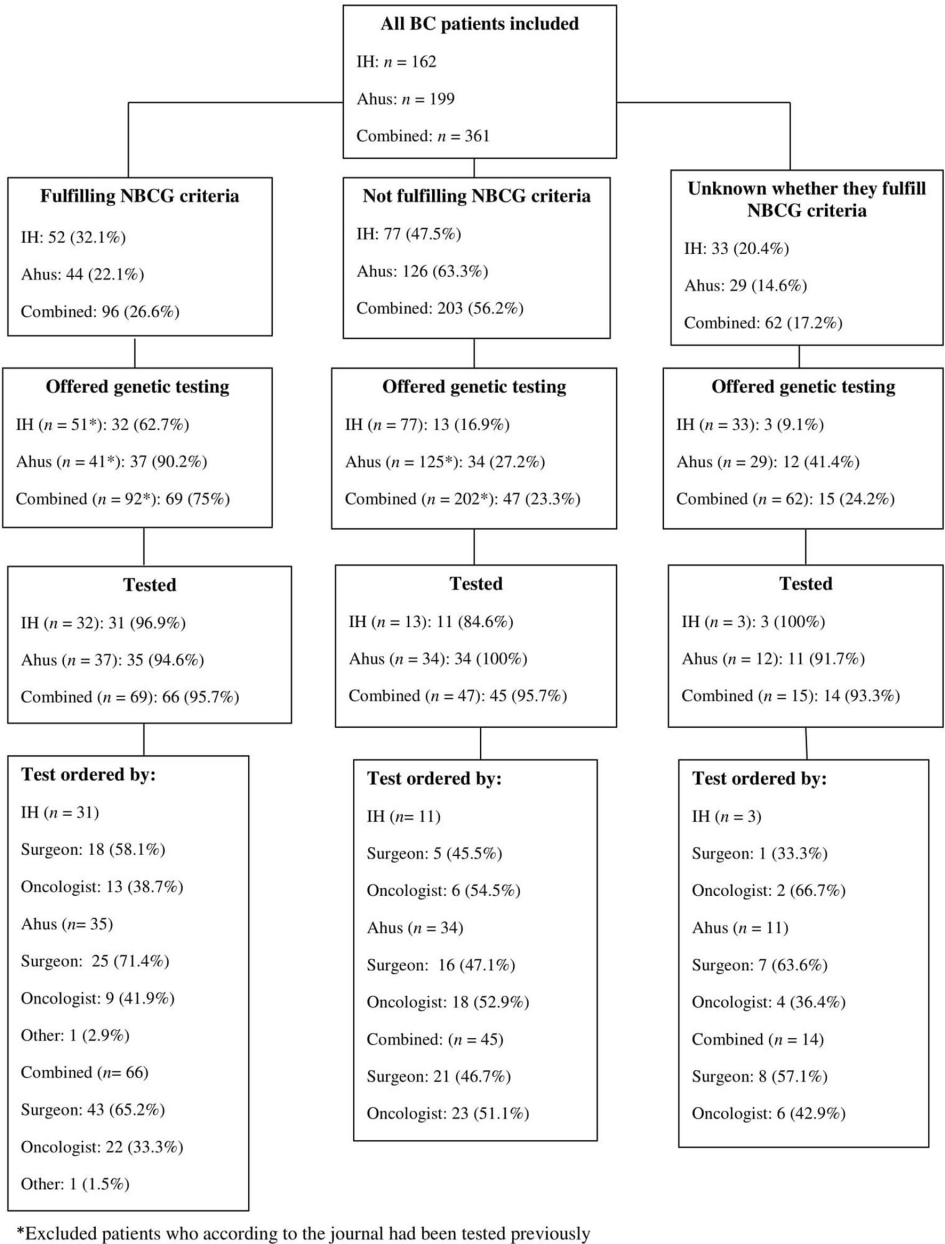
Genetic testing according to the criteria issued by the Norwegian Breast Cancer Group

The results regarding genetic testing according to whether or not the patient fulfilled the NBCG criteria can be found in Fig. 1. In total, 96/361 (26.6%) patients fulfilled the NBCG criteria. Four of these had been tested previously. Excluding these, 69/92 (75%) of BC patients who fulfilled the criteria were offered testing, 32/51 (62.7%) at IH and 37/41 (90.2%) at Ahus. At IH 18/31 (58.1%) had been tested by their surgeon, and 12/31 (38.7%) by their oncologist, while at Ahus, 25/35 (71.4%) were tested by their surgeon and 9/35 (25.7%) by their oncologist.

Scoring each of the 92 patients who fulfilled the NBCG criteria according to what part of the criteria they fulfilled, and according to whether or not they had been offered testing, 19 out of 29 patients (65.2%) diagnosed with BC below 50 years at IH had been offered testing and 28/30 (93.5%) at Ahus. Of the patients who were 50 years or older at time of diagnosis, and fulfilled the criteria only because they had a family history of BC and/or OC, 10/17 (58.8%) were offered testing at IH and 5/5 at Ahus. None of the two men with BC were offered testing (Table 4). Twelve of the 23 patients who fulfilled the criteria but were not offered testing (52.2%) were under 50 years at time of diagnosis (Table 5).

**Table 4:** Number of BC patients offered testing according to what part of the NBCG criteria they fulfill

Criteria	Innlandet Hospital	Ahus	Combined
BC < 50 years	19/29 (65.2%)	28/30 (93.5%)	47/59 (79.7%)
Bilateral BC < 60 years	2/2	1/1	3/3
TNBC < 60 years	1/3	3/3	4/6 (66.7%)
Family history of BC and/or OC*	10/17 (58.8%)	5/5	15/22 (68.2%)
Male breast cancer	–	0/2	0/2

*These patients were 50 years or above at time of diagnosis, and did not fulfill any of the other criteria (TNBC < 60 years, bilateral BC < 60 years or male BC

**Table 5:** Characteristics of BC patients fulfilling criteria who were not offered testing

Criteria fulfilled	Patients (n = 23)
BC < 50	12 (52.2%)
TNBC < 60	2 (8.7%)
Family history of BC and/or OC*	7 (30.4%)
Male BC	2 (8.7%)

*These patients were 50 years or above at time of diagnosis, and did not fulfill any of the other criteria (TNBC < 60 years, bilateral BC < 60 years or male BC)

## Discussion

This study is the first report of rates of genetic testing in Norway. It includes observations from two hospitals and may provide important insight for the continuous work of making this health service available for all eligible BC patients. The main objective was to investigate to what extent BC patients who fulfill the NBCG criteria were offered genetic testing, and we found that 75% of these patients were offered testing. Other studies have reported testing rates ranging from 15.3% to 60% [16–20]. The design of our study does not enable us to fully explain why we have observed such a high rate of testing compared to previous studies. We suspect that it may be due to the increasing awareness and availability of testing during the last 10 years, but also to the fact that genetic testing has been mainstreamed into regular oncological care in South Eastern Norway since 2014.

Even though the majority of eligible patients were offered testing during the study period, we observed differences between the hospitals, as 63% of eligible patients were offered testing at the regional hospital compared to 90% at the university hospital. The lower rates were observed both for the patients who fulfilled the criteria due to young age of onset and the older patients who had a family history of cancer. Our data do not provide systematic information on why there was a difference between the two hospitals. At the university hospital, the EPR had a standardized format with headings that included the term “heredity”, whereas the EPR at the regional hospital to a lesser degree seemed to have a set structure with pre-defined headings. Having a set structure with headings will remind the clinician of asking about family history and may also remind the clinicians of genetic testing. The observed difference may also be due to differences in awareness and traditions regarding genetic testing between the two hospitals.

We have previously estimated that 39% of all BC patients in South Eastern Norway were tested in 2014 and 2015 [21]. In the current study we found that 36.8% of all BC patients (29.8% at IH and 42.6% at Ahus) were offered testing, and that 34.6% (27.8% at IH and 40.2% at Ahus) had been tested. The estimate from 2014 and 2015 covers all hospitals in South Eastern Norway (except from OUH) and numbers may vary between hospitals. Comparisons should therefore be made with caution, but the overall rates of testing may not have increased significantly since then.

For the two hospitals combined, as many as 52% of the patients eligible for testing according to the NBCG criteria who had not been offered testing, were young at time of diagnosis (below 50 years). Our data are based on information registered in the EPR. We cannot exclude that some of these patients have been offered testing and declined, without it being noted. We also do not know whether they have a different demographic profile in terms of for example education level or marriage status compared to those that were tested. Nevertheless, our observations indicate that, at the moment, not all young BC patients are offered testing and there may be discrepancies between hospitals in the extent to which the health service reaches this group of patients. Studies have shown that 5–10% of BC patients below 50 years have a pathogenic *BRCA* variant [21, 24]. Young carriers have many years ahead with an increased risk of contralateral BC and OC. They are the ones who will benefit the most from cancer prevention and hence, genetic testing. It is therefore important that routines for genetic testing ensure that these patients have access to this health service.

There were only two male BC patients in our cohort, but none of them were offered testing. We cannot generalize based on these two men, but it should be emphasized in the guidelines that male BC patients have an approximately 10% risk of carrying a germline pathogenic *BRCA* variant, and should therefore be offered testing [reviewed in 25].

As many as 95% of the patients who were offered testing wanted to be tested, demonstrating that this a health service that BC patients want. In contrast, in the DNA-BONus study, only 45.4% of BC patients who were offered testing completed the test. In this study, all breast and ovarian cancer patients diagnosed between 2012 and 2015 at hospitals in the western part of Norway were offered *BRCA* testing as part of a research project [26]. We cannot rule out that there are some selection biases in our study. Apart from this the difference might be explained by the already mentioned increasing awareness of hereditary breast and ovarian cancer among cancer patients during the last years, and especially after Angelina Jolie shared her story in May 2013 [27].

At Ahus and IH, 57% of all tests were requested by the surgeon at time of diagnosis, and 42% by the oncologist during chemotherapy. It is not stated in the guidelines when genetic testing should be done, but because the majority of patients meet the surgeon first, there is an understanding that it is the surgeons who bear the main responsibility. It is therefore surprising that 42% of the patients were offered testing by their oncologist. Based on our findings we argue that there is a need for definitions and guidelines regarding when genetic testing should be offered, and also to ensure that patients who do not undergo chemotherapy are offered testing.

According to the current NBCG criteria, the treating physician can offer BC patients testing of the two *BRCA* genes only. If the patients’ personal or family history of cancer indicates that other genes could be relevant, the patient should be referred to genetic counseling [13]. Several studies have reported that offering BC patients testing of multi gene panels results in clinical significant findings in other breast cancer genes [28–31]. Whether surgeons and oncologists also should offer multi gene panels to BC patients is a continuous discussion in Norway. Given that not all eligible patients are offered testing today, such an expansion may require more training in genetics for the health personnel involved in diagnostics and treatment of BC.

There are some limitations to our study. Firstly, our data are based on information from patient records alone, and we cannot rule out that there may be information relevant for the study that was not recorded here. Some patients may have been offered but have declined testing, without it being noted, and family history could have been asked, but not registered. In addition, we did not collect any demographic data on the patients. The aim of this study was to investigate to what extent the health care system is able to offer genetic testing to BC patients according to clinical guidelines. It is a small study, including data from only two hospitals, and demographic factors were considered to be less relevant. Some studies have demonstrated that factors such as ethnicity, education level and income may affect rates of referral to genetic counseling and testing [32–35]. These studies have not been restricted to newly diagnosed BC patients. Other studies have reported that access to testing is more affected by barriers on a provider or system level [36–39].We do not know whether the eligible patients that were not offered testing had a different demographic profile than the patients who were offered testing. Based on the information that was found in the EPR, we registered whether testing had been initiated by the physician or the patient. For all but three of all tested patients, the physician had initiated the discussion of testing, indicating that demographic factors could be of limited importance for those who did not get access to testing, but we cannot confirm this. The response rate for inclusion in the study and access to medical records was 63.3% for the regional hospital and 65.7% for the university hospital. No second reminder was sent to the patients. We only have information about those who responded. Even if the response rate was similar for the two hospitals, we cannot exclude that the dataset may be skewed either towards those who have been offered testing and/or have accepted testing, towards those who were not offered testing and/or did not go through with testing or affected by demographic factors. However, the two cohorts were similar in terms of age distribution, and also similar to the age distribution of all BC patients in Norway [40].

## Conclusions

In conclusion, in two hospitals in South Eastern Norway where diagnostic genetic testing is offered directly to BC patients by their surgeon or oncologist, the majority of patients who fulfilled the criteria were offered testing. The design of the study does not allow us to fully explain why the rates are higher than what has been observed in other studies. We nevertheless suspect that mainstreaming genetic testing into regular oncological care has contributed. The high rates of testing therefore support this change of practice. However, there were important differences in rates of testing between the hospitals that affected all groups of patients. This indicates that diagnostic genetic testing of BC patients is not equally available to all patients. We have also observed that 95% of BC patients who were offered testing wanted to be tested. Based on our findings we therefore suggest that efforts should be made to increase awareness and knowledge of, and improve routines for genetic testing among clinicians that in turn will contribute to make genetic testing an integral part of diagnosis and treatment of BC in Norway.

## Abbreviations

Ahus: Akershus University Hospital
BC: Breast cancer
EPR: Electronic patient record
IH: Innlandet Hospital
NBCG: Norwegian Breast Cancer Group
OC: Ovarian cancer
OUH: Oslo University Hospital
TNBC: Triple negative breast cancer
TSD: Service for sensitive data
VUS: Variant of unknown clinical significance
